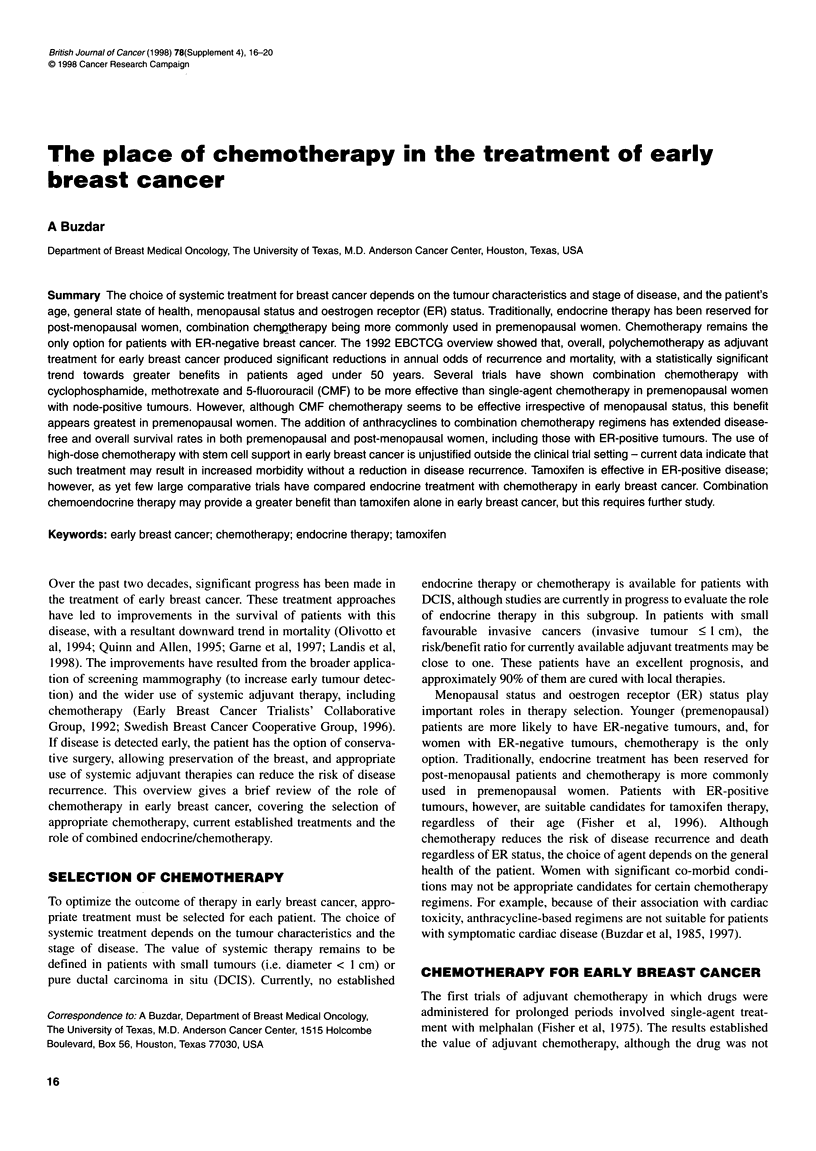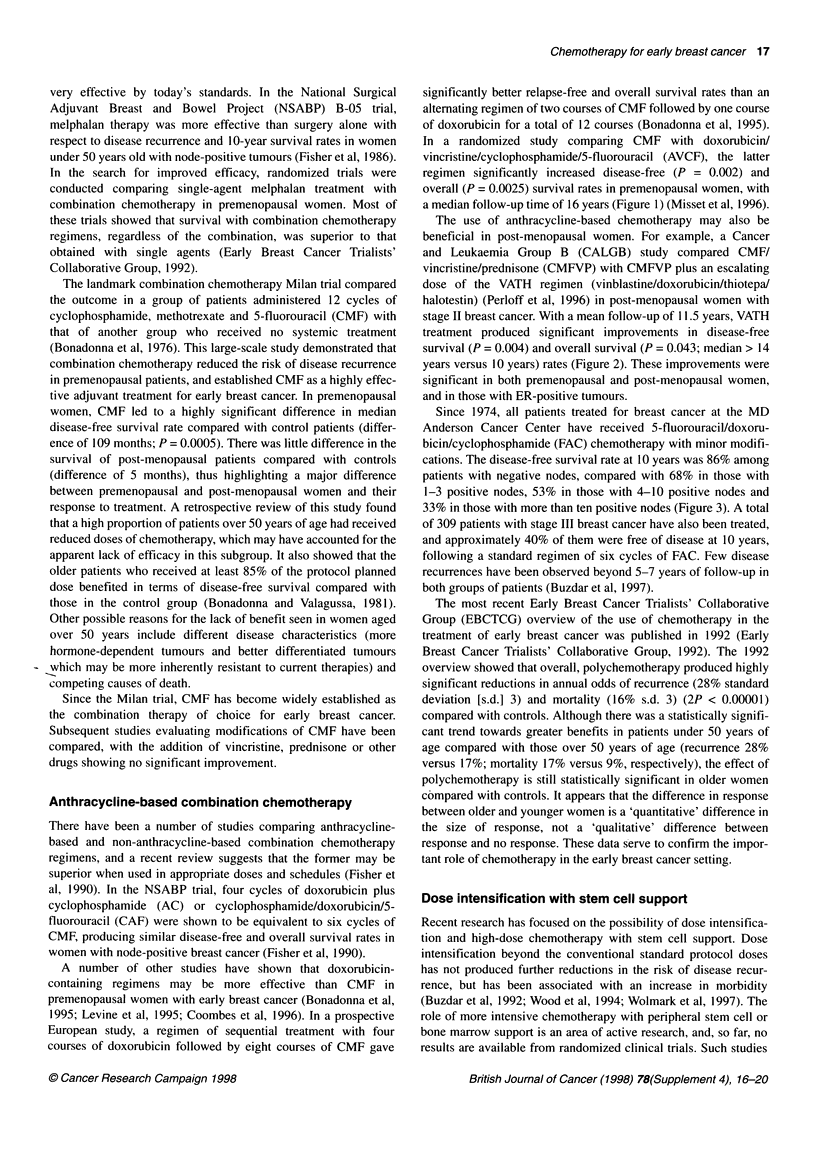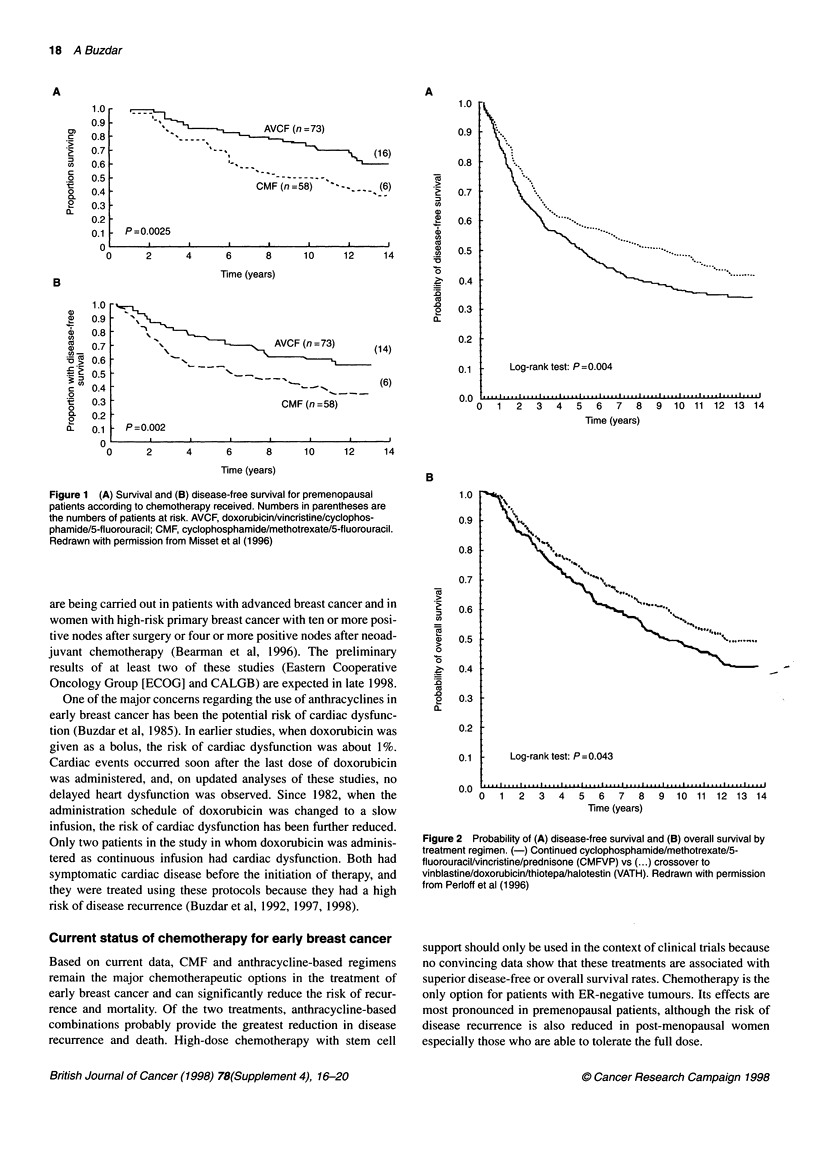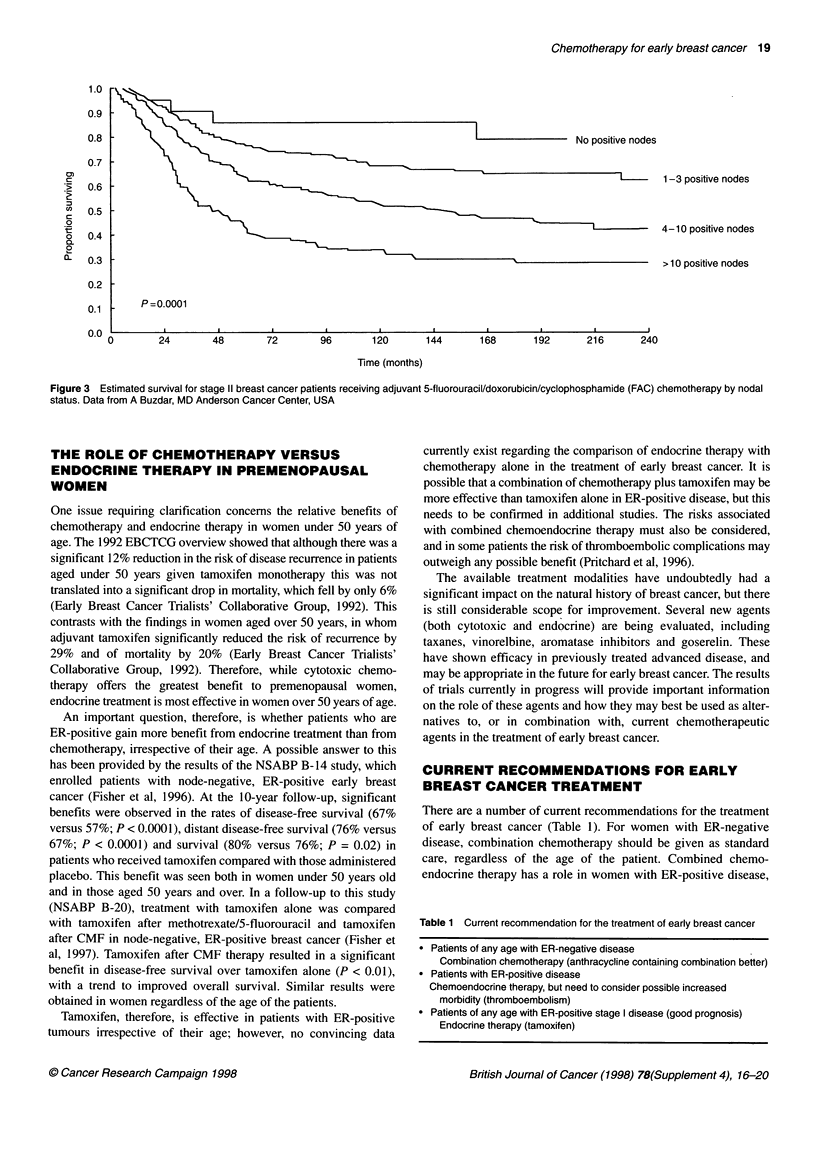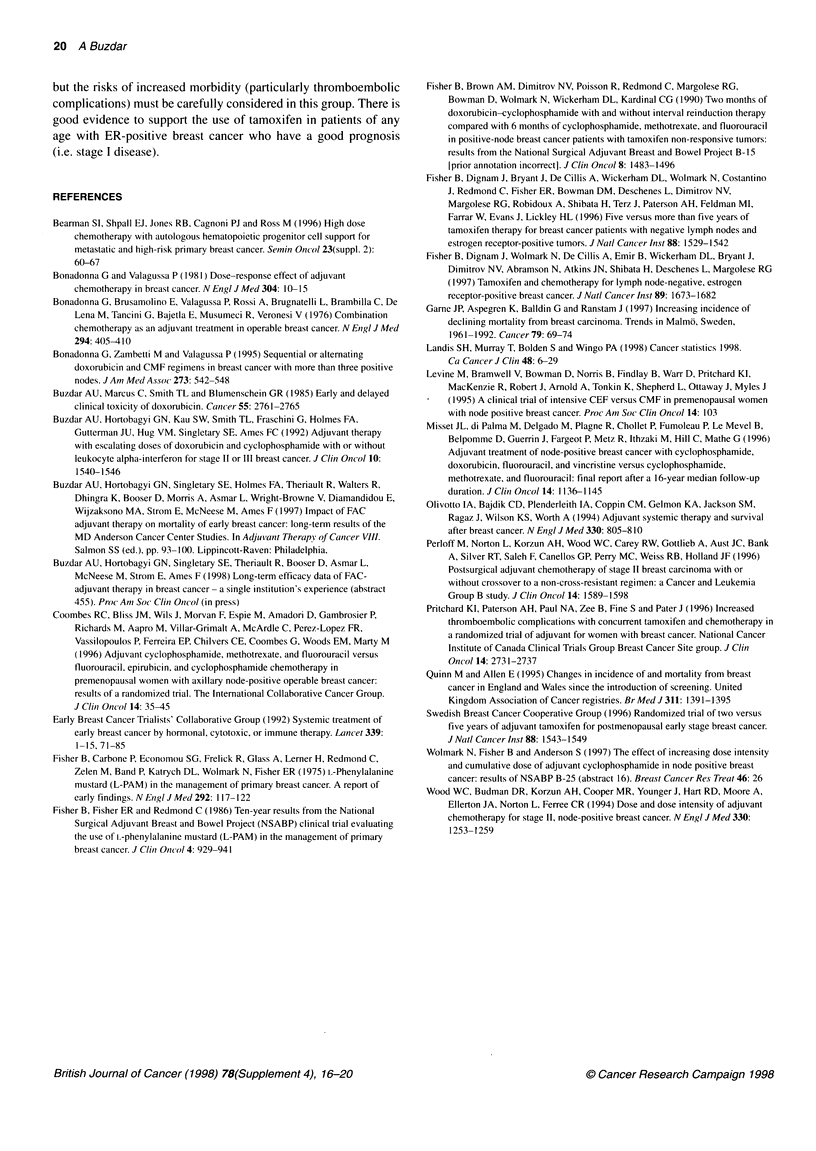# The place of chemotherapy in the treatment of early breast cancer.

**DOI:** 10.1038/bjc.1998.757

**Published:** 1998-09

**Authors:** A. Buzdar

**Affiliations:** Department of Breast Medical Oncology, The University of Texas, M.D. Anderson Cancer Center, Houston 77030, USA.

## Abstract

The choice of systemic treatment for breast cancer depends on the tumour characteristics and stage of disease, and the patient's age, general state of health, menopausal status and oestrogen receptor (ER) status. Traditionally, endocrine therapy has been reserved for post-menopausal women, combination chemotherapy being more commonly used in premenopausal women. Chemotherapy remains the only option for patients with ER-negative breast cancer. The 1992 EBCTCG overview showed that, overall, polychemotherapy as adjuvant treatment for early breast cancer produced significant reductions in annual odds of recurrence and mortality, with a statistically significant trend towards greater benefits in patients aged under 50 years. Several trials have shown combination chemotherapy with cyclophosphamide, methotrexate and 5-fluorouracil (CMF) to be more effective than single-agent chemotherapy in premenopausal women with node-positive tumours. However, although CMF chemotherapy seems to be effective irrespective of menopausal status, this benefit appears greatest in premenopausal women. The addition of anthracyclines to combination chemotherapy regimens has extended disease-free and overall survival rates in both premenopausal and post-menopausal women, including those with ER-positive tumours. The use of high-dose chemotherapy with stem cell support in early breast cancer is unjustified outside the clinical trial setting--current data indicate that such treatment may result in increased morbidity without a reduction in disease recurrence. Tamoxifen is effective in ER-positive disease; however, as yet few large comparative trials have compared endocrine treatment with chemotherapy in early breast cancer. Combination chemoendocrine therapy may provide a greater benefit than tamoxifen alone in early breast cancer, but this requires further study.


British Journal of Cancer (1998) 78(Supplement 4), 16–20
© 1998 Cancer Research Campaign

### The place of chemotherapy in the treatment of early breast cancer

**A Buzdar**

Department of Breast Medical Oncology, The University of Texas, M.D. Anderson Cancer Center, Houston, Texas, USA

**Summary** The choice of systemic treatment for breast cancer depends on the tumour characteristics and stage of disease, and the patient's age, general state of health, menopausal status and oestrogen receptor (ER) status. Traditionally, endocrine therapy has been reserved for post-menopausal women, combination chemotherapy being more commonly used in premenopausal women. Chemotherapy remains the only option for patients with ER-negative breast cancer. The 1992 EBCTCG overview showed that, overall, polychemotherapy as adjuvant treatment for early breast cancer produced significant reductions in annual odds of recurrence and mortality, with a statistically significant trend towards greater benefits in patients aged under 50 years. Several trials have shown combination chemotherapy with cyclophosphamide, methotrexate and 5-fluorouracil (CMF) to be more effective than single-agent chemotherapy in premenopausal women with node-positive tumours. However, although CMF chemotherapy seems to be effective irrespective of menopausal status, this benefit appears greatest in premenopausal women. The addition of anthracyclines to combination chemotherapy regimens has extended disease-free and overall survival rates in both premenopausal and post-menopausal women, including those with ER-positive tumours. The use of high-dose chemotherapy with stem cell support in early breast cancer is unjustified outside the clinical trial setting – current data indicate that such treatment may result in increased morbidity without a reduction in disease recurrence. Tamoxifen is effective in ER-positive disease; however, as yet few large comparative trials have compared endocrine treatment with chemotherapy in early breast cancer. Combination chemoendocrine therapy may provide a greater benefit than tamoxifen alone in early breast cancer, but this requires further study.

**Keywords:** early breast cancer; chemotherapy; endocrine therapy; tamoxifen

Over the past two decades, significant progress has been made in the treatment of early breast cancer. These treatment approaches have led to improvements in the survival of patients with this disease, with a resultant downward trend in mortality (Olivotto et al, 1994; Quinn and Allen, 1995; Garne et al, 1997; Landis et al, 1998). The improvements have resulted from the broader application of screening mammography (to increase early tumour detection) and the wider use of systemic adjuvant therapy, including chemotherapy (Early Breast Cancer Trialists' Collaborative Group, 1992; Swedish Breast Cancer Cooperative Group, 1996). If disease is detected early, the patient has the option of conservative surgery, allowing preservation of the breast, and appropriate use of systemic adjuvant therapies can reduce the risk of disease recurrence. This overview gives a brief review of the role of chemotherapy in early breast cancer, covering the selection of appropriate chemotherapy, current established treatments and the role of combined endocrine/chemotherapy.

### SELECTION OF CHEMOTHERAPY

To optimize the outcome of therapy in early breast cancer, appropriate treatment must be selected for each patient. The choice of systemic treatment depends on the tumour characteristics and the stage of disease. The value of systemic therapy remains to be defined in patients with small tumours (i.e. diameter < 1 cm) or pure ductal carcinoma in situ (DCIS). Currently, no established endocrine therapy or chemotherapy is available for patients with DCIS, although studies are currently in progress to evaluate the role of endocrine therapy in this subgroup. In patients with small favourable invasive cancers (invasive tumour ≤ 1 cm), the risk/benefit ratio for currently available adjuvant treatments may be close to one. These patients have an excellent prognosis, and approximately 90% of them are cured with local therapies.

Menopausal status and oestrogen receptor (ER) status play important roles in therapy selection. Younger (premenopausal) patients are more likely to have ER-negative tumours, and, for women with ER-negative tumours, chemotherapy is the only option. Traditionally, endocrine treatment has been reserved for post-menopausal patients and chemotherapy is more commonly used in premenopausal women. Patients with ER-positive tumours, however, are suitable candidates for tamoxifen therapy, regardless of their age (Fisher et al, 1996). Although chemotherapy reduces the risk of disease recurrence and death regardless of ER status, the choice of agent depends on the general health of the patient. Women with significant co-morbid conditions may not be appropriate candidates for certain chemotherapy regimens. For example, because of their association with cardiac toxicity, anthracycline-based regimens are not suitable for patients with symptomatic cardiac disease (Buzdar et al, 1985, 1997).

### CHEMOTHERAPY FOR EARLY BREAST CANCER

The first trials of adjuvant chemotherapy in which drugs were administered for prolonged periods involved single-agent treatment with melphalan (Fisher et al, 1975). The results established the value of adjuvant chemotherapy, although the drug was not

*Correspondence to:* A Buzdar, Department of Breast Medical Oncology, The University of Texas, M.D. Anderson Cancer Center, 1515 Holcombe Boulevard, Box 56, Houston, Texas 77030, USA

very effective by today's standards. In the National Surgical Adjuvant Breast and Bowel Project (NSABP) B-05 trial, melphalan therapy was more effective than surgery alone with respect to disease recurrence and 10-year survival rates in women under 50 years old with node-positive tumours (Fisher et al, 1986). In the search for improved efficacy, randomized trials were conducted comparing single-agent melphalan treatment with combination chemotherapy in premenopausal women. Most of these trials showed that survival with combination chemotherapy regimens, regardless of the combination, was superior to that obtained with single agents (Early Breast Cancer Trialists' Collaborative Group, 1992).

The landmark combination chemotherapy Milan trial compared the outcome in a group of patients administered 12 cycles of cyclophosphamide, methotrexate and 5-fluorouracil (CMF) with that of another group who received no systemic treatment (Bonadonna et al, 1976). This large-scale study demonstrated that combination chemotherapy reduced the risk of disease recurrence in premenopausal patients, and established CMF as a highly effective adjuvant treatment for early breast cancer. In premenopausal women, CMF led to a highly significant difference in median disease-free survival rate compared with control patients (difference of 109 months; $P = 0.0005$). There was little difference in the survival of post-menopausal patients compared with controls (difference of 5 months), thus highlighting a major difference between premenopausal and post-menopausal women and their response to treatment. A retrospective review of this study found that a high proportion of patients over 50 years of age had received reduced doses of chemotherapy, which may have accounted for the apparent lack of efficacy in this subgroup. It also showed that the older patients who received at least 85% of the protocol planned dose benefited in terms of disease-free survival compared with those in the control group (Bonadonna and Valagussa, 1981). Other possible reasons for the lack of benefit seen in women aged over 50 years include different disease characteristics (more hormone-dependent tumours and better differentiated tumours which may be more inherently resistant to current therapies) and competing causes of death.

Since the Milan trial, CMF has become widely established as the combination therapy of choice for early breast cancer. Subsequent studies evaluating modifications of CMF have been compared, with the addition of vincristine, prednisone or other drugs showing no significant improvement.

### Anthracycline-based combination chemotherapy

There have been a number of studies comparing anthracycline-based and non-anthracycline-based combination chemotherapy regimens, and a recent review suggests that the former may be superior when used in appropriate doses and schedules (Fisher et al, 1990). In the NSABP trial, four cycles of doxorubicin plus cyclophosphamide (AC) or cyclophosphamide/doxorubicin/5-fluorouracil (CAF) were shown to be equivalent to six cycles of CMF, producing similar disease-free and overall survival rates in women with node-positive breast cancer (Fisher et al, 1990).

A number of other studies have shown that doxorubicin-containing regimens may be more effective than CMF in premenopausal women with early breast cancer (Bonadonna et al, 1995; Levine et al, 1995; Coombes et al, 1996). In a prospective European study, a regimen of sequential treatment with four courses of doxorubicin followed by eight courses of CMF gave significantly better relapse-free and overall survival rates than an alternating regimen of two courses of CMF followed by one course of doxorubicin for a total of 12 courses (Bonadonna et al, 1995). In a randomized study comparing CMF with doxorubicin/vincristine/cyclophosphamide/5-fluorouracil (AVCF), the latter regimen significantly increased disease-free ($P = 0.002$) and overall ($P = 0.0025$) survival rates in premenopausal women, with a median follow-up time of 16 years (Figure 1) (Misset et al, 1996).

The use of anthracycline-based chemotherapy may also be beneficial in post-menopausal women. For example, a Cancer and Leukaemia Group B (CALGB) study compared CMF/vincristine/prednisone (CMFVP) with CMFVP plus an escalating dose of the VATH regimen (vinblastine/doxorubicin/thiotepa/halotestin) (Perloff et al, 1996) in post-menopausal women with stage II breast cancer. With a mean follow-up of 11.5 years, VATH treatment produced significant improvements in disease-free survival ($P = 0.004$) and overall survival ($P = 0.043$; median > 14 years versus 10 years) rates (Figure 2). These improvements were significant in both premenopausal and post-menopausal women, and in those with ER-positive tumours.

Since 1974, all patients treated for breast cancer at the MD Anderson Cancer Center have received 5-fluorouracil/doxorubicin/cyclophosphamide (FAC) chemotherapy with minor modifications. The disease-free survival rate at 10 years was 86% among patients with negative nodes, compared with 68% in those with 1–3 positive nodes, 53% in those with 4–10 positive nodes and 33% in those with more than ten positive nodes (Figure 3). A total of 309 patients with stage III breast cancer have also been treated, and approximately 40% of them were free of disease at 10 years, following a standard regimen of six cycles of FAC. Few disease recurrences have been observed beyond 5–7 years of follow-up in both groups of patients (Buzdar et al, 1997).

The most recent Early Breast Cancer Trialists' Collaborative Group (EBCTCG) overview of the use of chemotherapy in the treatment of early breast cancer was published in 1992 (Early Breast Cancer Trialists' Collaborative Group, 1992). The 1992 overview showed that overall, polychemotherapy produced highly significant reductions in annual odds of recurrence (28% standard deviation [s.d.] 3) and mortality (16% s.d. 3) ($2P < 0.00001$) compared with controls. Although there was a statistically significant trend towards greater benefits in patients under 50 years of age compared with those over 50 years of age (recurrence 28% versus 17%; mortality 17% versus 9%, respectively), the effect of polychemotherapy is still statistically significant in older women compared with controls. It appears that the difference in response between older and younger women is a 'quantitative' difference in the size of response, not a 'qualitative' difference between response and no response. These data serve to confirm the important role of chemotherapy in the early breast cancer setting.

### Dose intensification with stem cell support

Recent research has focused on the possibility of dose intensification and high-dose chemotherapy with stem cell support. Dose intensification beyond the conventional standard protocol doses has not produced further reductions in the risk of disease recurrence, but has been associated with an increase in morbidity (Buzdar et al, 1992; Wood et al, 1994; Wolmark et al, 1997). The role of more intensive chemotherapy with peripheral stem cell or bone marrow support is an area of active research, and, so far, no results are available from randomized clinical trials. Such studies

© Cancer Research Campaign 1998 

**Figure 1** (**A**) Survival and (**B**) disease-free survival for premenopausal patients according to chemotherapy received. Numbers in parentheses are the numbers of patients at risk. AVCF, doxorubicin/vincristine/cyclophosphamide/5-fluorouracil; CMF, cyclophosphamide/methotrexate/5-fluorouracil. Redrawn with permission from Misset et al (1996)

**Figure 2** Probability of (**A**) disease-free survival and (**B**) overall survival by treatment regimen. (—) Continued cyclophosphamide/methotrexate/5-fluorouracil/vincristine/prednisone (CMFVP) vs (...) crossover to vinblastine/doxorubicin/thiotepa/halotestin (VATH). Redrawn with permission from Perloff et al (1996)

are being carried out in patients with advanced breast cancer and in women with high-risk primary breast cancer with ten or more positive nodes after surgery or four or more positive nodes after neoadjuvant chemotherapy (Bearman et al, 1996). The preliminary results of at least two of these studies (Eastern Cooperative Oncology Group [ECOG] and CALGB) are expected in late 1998.

One of the major concerns regarding the use of anthracyclines in early breast cancer has been the potential risk of cardiac dysfunction (Buzdar et al, 1985). In earlier studies, when doxorubicin was given as a bolus, the risk of cardiac dysfunction was about 1%. Cardiac events occurred soon after the last dose of doxorubicin was administered, and, on updated analyses of these studies, no delayed heart dysfunction was observed. Since 1982, when the administration schedule of doxorubicin was changed to a slow infusion, the risk of cardiac dysfunction has been further reduced. Only two patients in the study in whom doxorubicin was administered as continuous infusion had cardiac dysfunction. Both had symptomatic cardiac disease before the initiation of therapy, and they were treated using these protocols because they had a high risk of disease recurrence (Buzdar et al, 1992, 1997, 1998).

### Current status of chemotherapy for early breast cancer

Based on current data, CMF and anthracycline-based regimens remain the major chemotherapeutic options in the treatment of early breast cancer and can significantly reduce the risk of recurrence and mortality. Of the two treatments, anthracycline-based combinations probably provide the greatest reduction in disease recurrence and death. High-dose chemotherapy with stem cell support should only be used in the context of clinical trials because no convincing data show that these treatments are associated with superior disease-free or overall survival rates. Chemotherapy is the only option for patients with ER-negative tumours. Its effects are most pronounced in premenopausal patients, although the risk of disease recurrence is also reduced in post-menopausal women especially those who are able to tolerate the full dose.

© Cancer Research Campaign 1998

**Figure 3** Estimated survival for stage II breast cancer patients receiving adjuvant 5-fluorouracil/doxorubicin/cyclophosphamide (FAC) chemotherapy by nodal status. Data from A Buzdar, MD Anderson Cancer Center, USA

### THE ROLE OF CHEMOTHERAPY VERSUS ENDOCRINE THERAPY IN PREMENOPAUSAL WOMEN

One issue requiring clarification concerns the relative benefits of chemotherapy and endocrine therapy in women under 50 years of age. The 1992 EBCTCG overview showed that although there was a significant 12% reduction in the risk of disease recurrence in patients aged under 50 years given tamoxifen monotherapy this was not translated into a significant drop in mortality, which fell by only 6% (Early Breast Cancer Trialists' Collaborative Group, 1992). This contrasts with the findings in women aged over 50 years, in whom adjuvant tamoxifen significantly reduced the risk of recurrence by 29% and of mortality by 20% (Early Breast Cancer Trialists' Collaborative Group, 1992). Therefore, while cytotoxic chemotherapy offers the greatest benefit to premenopausal women, endocrine treatment is most effective in women over 50 years of age.

An important question, therefore, is whether patients who are ER-positive gain more benefit from endocrine treatment than from chemotherapy, irrespective of their age. A possible answer to this has been provided by the results of the NSABP B-14 study, which enrolled patients with node-negative, ER-positive early breast cancer (Fisher et al, 1996). At the 10-year follow-up, significant benefits were observed in the rates of disease-free survival (67% versus 57%; $P < 0.0001$), distant disease-free survival (76% versus 67%; $P < 0.0001$) and survival (80% versus 76%; $P = 0.02$) in patients who received tamoxifen compared with those administered placebo. This benefit was seen both in women under 50 years old and in those aged 50 years and over. In a follow-up to this study (NSABP B-20), treatment with tamoxifen alone was compared with tamoxifen after methotrexate/5-fluorouracil and tamoxifen after CMF in node-negative, ER-positive breast cancer (Fisher et al, 1997). Tamoxifen after CMF therapy resulted in a significant benefit in disease-free survival over tamoxifen alone ($P < 0.01$), with a trend to improved overall survival. Similar results were obtained in women regardless of the age of the patients.

Tamoxifen, therefore, is effective in patients with ER-positive tumours irrespective of their age; however, no convincing data currently exist regarding the comparison of endocrine therapy with chemotherapy alone in the treatment of early breast cancer. It is possible that a combination of chemotherapy plus tamoxifen may be more effective than tamoxifen alone in ER-positive disease, but this needs to be confirmed in additional studies. The risks associated with combined chemoendocrine therapy must also be considered, and in some patients the risk of thromboembolic complications may outweigh any possible benefit (Pritchard et al, 1996).

The available treatment modalities have undoubtedly had a significant impact on the natural history of breast cancer, but there is still considerable scope for improvement. Several new agents (both cytotoxic and endocrine) are being evaluated, including taxanes, vinorelbine, aromatase inhibitors and goserelin. These have shown efficacy in previously treated advanced disease, and may be appropriate in the future for early breast cancer. The results of trials currently in progress will provide important information on the role of these agents and how they may best be used as alternatives to, or in combination with, current chemotherapeutic agents in the treatment of early breast cancer.

### CURRENT RECOMMENDATIONS FOR EARLY BREAST CANCER TREATMENT

There are a number of current recommendations for the treatment of early breast cancer (Table 1). For women with ER-negative disease, combination chemotherapy should be given as standard care, regardless of the age of the patient. Combined chemoendocrine therapy has a role in women with ER-positive disease,

**Table 1** Current recommendation for the treatment of early breast cancer

| |
|---|
| • Patients of any age with ER-negative disease<br>Combination chemotherapy (anthracycline containing combination better) |
| • Patients with ER-positive disease<br>Chemoendocrine therapy, but need to consider possible increased morbidity (thromboembolism) |
| • Patients of any age with ER-positive stage I disease (good prognosis)<br>Endocrine therapy (tamoxifen) |

but the risks of increased morbidity (particularly thromboembolic complications) must be carefully considered in this group. There is good evidence to support the use of tamoxifen in patients of any age with ER-positive breast cancer who have a good prognosis (i.e. stage I disease).

### REFERENCES

Bearman SI, Shpall EJ, Jones RB, Cagnoni PJ and Ross M (1996) High dose chemotherapy with autologous hematopoietic progenitor cell support for metastatic and high-risk primary breast cancer. *Semin Oncol* **23**(suppl. 2): 60–67

Bonadonna G and Valagussa P (1981) Dose–response effect of adjuvant chemotherapy in breast cancer. *N Engl J Med* **304**: 10–15

Bonadonna G, Brusamolino E, Valagussa P, Rossi A, Brugnatelli L, Brambilla C, De Lena M, Tancini G, Bajetla E, Musumeci R, Veronesi V (1976) Combination chemotherapy as an adjuvant treatment in operable breast cancer. *N Engl J Med* **294**: 405–410

Bonadonna G, Zambetti M and Valagussa P (1995) Sequential or alternating doxorubicin and CMF regimens in breast cancer with more than three positive nodes. *J Am Med Assoc* **273**: 542–548

Buzdar AU, Marcus C, Smith TL and Blumenschein GR (1985) Early and delayed clinical toxicity of doxorubicin. *Cancer* **55**: 2761–2765

Buzdar AU, Hortobagyi GN, Kau SW, Smith TL, Fraschini G, Holmes FA, Gutterman JU, Hug VM, Singletary SE, Ames FC (1992) Adjuvant therapy with escalating doses of doxorubicin and cyclophosphamide with or without leukocyte alpha-interferon for stage II or III breast cancer. *J Clin Oncol* **10**: 1540–1546

Buzdar AU, Hortobagyi GN, Singletary SE, Holmes FA, Theriault R, Walters R, Dhingra K, Booser D, Morris A, Asmar L, Wright-Browne V, Diamandidou E, Wijzaksono MA, Strom E, McNeese M, Ames F (1997) Impact of FAC adjuvant therapy on mortality of early breast cancer: long-term results of the MD Anderson Cancer Center Studies. In *Adjuvant Therapy of Cancer VIII*. Salmon SS (ed.), pp. 93–100. Lippincott-Raven: Philadelphia.

Buzdar AU, Hortobagyi GN, Singletary SE, Theriault R, Booser D, Asmar L, McNeese M, Strom E, Ames F (1998) Long-term efficacy data of FAC-adjuvant therapy in breast cancer – a single institution's experience (abstract 455). *Proc Am Soc Clin Oncol* (in press)

Coombes RC, Bliss JM, Wils J, Morvan F, Espie M, Amadori D, Gambrosier P, Richards M, Aapro M, Villar-Grimalt A, McArdle C, Perez-Lopez FR, Vassilopoulos P, Ferreira EP, Chilvers CE, Coombes G, Woods EM, Marty M (1996) Adjuvant cyclophosphamide, methotrexate, and fluorouracil versus fluorouracil, epirubicin, and cyclophosphamide chemotherapy in premenopausal women with axillary node-positive operable breast cancer: results of a randomized trial. The International Collaborative Cancer Group. *J Clin Oncol* **14**: 35–45

Early Breast Cancer Trialists' Collaborative Group (1992) Systemic treatment of early breast cancer by hormonal, cytotoxic, or immune therapy. *Lancet* **339**: 1–15, 71–85

Fisher B, Carbone P, Economou SG, Frelick R, Glass A, Lerner H, Redmond C, Zelen M, Band P, Katrych DL, Wolmark N, Fisher ER (1975) L-Phenylalanine mustard (L-PAM) in the management of primary breast cancer. A report of early findings. *N Engl J Med* **292**: 117–122

Fisher B, Fisher ER and Redmond C (1986) Ten-year results from the National Surgical Adjuvant Breast and Bowel Project (NSABP) clinical trial evaluating the use of L-phenylalanine mustard (L-PAM) in the management of primary breast cancer. *J Clin Oncol* **4**: 929–941

Fisher B, Brown AM, Dimitrov NV, Poisson R, Redmond C, Margolese RG, Bowman D, Wolmark N, Wickerham DL, Kardinal CG (1990) Two months of doxorubicin–cyclophosphamide with and without interval reinduction therapy compared with 6 months of cyclophosphamide, methotrexate, and fluorouracil in positive-node breast cancer patients with tamoxifen non-responsive tumors: results from the National Surgical Adjuvant Breast and Bowel Project B-15 [prior annotation incorrect]. *J Clin Oncol* **8**: 1483–1496

Fisher B, Dignam J, Bryant J, De Cillis A, Wickerham DL, Wolmark N, Costantino J, Redmond C, Fisher ER, Bowman DM, Deschenes L, Dimitrov NV, Margolese RG, Robidoux A, Shibata H, Terz J, Paterson AH, Feldman MI, Farrar W, Evans J, Lickley HL (1996) Five versus more than five years of tamoxifen therapy for breast cancer patients with negative lymph nodes and estrogen receptor-positive tumors. *J Natl Cancer Inst* **88**: 1529–1542

Fisher B, Dignam J, Wolmark N, De Cillis A, Emir B, Wickerham DL, Bryant J, Dimitrov NV, Abramson N, Atkins JN, Shibata H, Deschenes L, Margolese RG (1997) Tamoxifen and chemotherapy for lymph node-negative, estrogen receptor-positive breast cancer. *J Natl Cancer Inst* **89**: 1673–1682

Garne JP, Aspegren K, Balldin G and Ranstam J (1997) Increasing incidence of declining mortality from breast carcinoma. Trends in Malmö, Sweden, 1961–1992. *Cancer* **79**: 69–74

Landis SH, Murray T, Bolden S and Wingo PA (1998) Cancer statistics 1998. *Ca Cancer J Clin* **48**: 6–29

Levine M, Bramwell V, Bowman D, Norris B, Findlay B, Warr D, Pritchard KI, MacKenzie R, Robert J, Arnold A, Tonkin K, Shepherd L, Ottaway J, Myles J (1995) A clinical trial of intensive CEF versus CMF in premenopausal women with node positive breast cancer. *Proc Am Soc Clin Oncol* **14**: 103

Misset JL, di Palma M, Delgado M, Plagne R, Chollet P, Fumoleau P, Le Mevel B, Belpomme D, Guerrin J, Fargeot P, Metz R, Ithzaki M, Hill C, Mathe G (1996) Adjuvant treatment of node-positive breast cancer with cyclophosphamide, doxorubicin, fluorouracil, and vincristine versus cyclophosphamide, methotrexate, and fluorouracil: final report after a 16-year median follow-up duration. *J Clin Oncol* **14**: 1136–1145

Olivotto IA, Bajdik CD, Plenderleith IA, Coppin CM, Gelmon KA, Jackson SM, Ragaz J, Wilson KS, Worth A (1994) Adjuvant systemic therapy and survival after breast cancer. *N Engl J Med* **330**: 805–810

Perloff M, Norton L, Korzun AH, Wood WC, Carey RW, Gottlieb A, Aust JC, Bank A, Silver RT, Saleh F, Canellos GP, Perry MC, Weiss RB, Holland JF (1996) Postsurgical adjuvant chemotherapy of stage II breast carcinoma with or without crossover to a non-cross-resistant regimen: a Cancer and Leukemia Group B study. *J Clin Oncol* **14**: 1589–1598

Pritchard KI, Paterson AH, Paul NA, Zee B, Fine S and Pater J (1996) Increased thromboembolic complications with concurrent tamoxifen and chemotherapy in a randomized trial of adjuvant for women with breast cancer. National Cancer Institute of Canada Clinical Trials Group Breast Cancer Site group. *J Clin Oncol* **14**: 2731–2737

Quinn M and Allen E (1995) Changes in incidence of and mortality from breast cancer in England and Wales since the introduction of screening. United Kingdom Association of Cancer registries. *Br Med J* **311**: 1391–1395

Swedish Breast Cancer Cooperative Group (1996) Randomized trial of two versus five years of adjuvant tamoxifen for postmenopausal early stage breast cancer. *J Natl Cancer Inst* **88**: 1543–1549

Wolmark N, Fisher B and Anderson S (1997) The effect of increasing dose intensity and cumulative dose of adjuvant cyclophosphamide in node positive breast cancer: results of NSABP B-25 (abstract 16). *Breast Cancer Res Treat* **46**: 26

Wood WC, Budman DR, Korzun AH, Cooper MR, Younger J, Hart RD, Moore A, Ellerton JA, Norton L, Ferree CR (1994) Dose and dose intensity of adjuvant chemotherapy for stage II, node-positive breast cancer. *N Engl J Med* **330**: 1253–1259

© Cancer Research Campaign 1998